# Supplementary material for: Chromothripsis during telomere crisis is independent of NHEJ, and consistent with a replicative origin
Source: Genome Res. 2019 May;29(5):737–49. doi: 10.1101/gr.240705.118 (PMC6499312; doi:10.1101/gr.240705.118)
Supplement: Supplemental Material [file supp_gr.240705.118_Supplemental_file_1.zip › contigs/annotated_contigs/DB115/contig.6.DB115_length_865_mean_cov_11.6184971098.docx]

**DB115_length_865_mean_cov_11.6184971098**

```
TGGGAATATAACAAAANCATCTGTGGTATACTAAATGAATTAACAACAATAAAAATGACAACAAATCCCTGAGATAAACAAACAAGATC
 >chr4:93022429-93022571 - E=2e-70
TAAGCTGACAACCACATAGGAATTATGTAAAGAGCAGTAATATCTGATGCA|GA|TAAGGCATGTTTCTCTGGCAGG|GA|AAGGGAGT
                                                    >chr4:93022332-93022358 + E=9e-04
TATTAAACAATCCCCATGACTGGTTAGCAATTCTCTCTTTAAGTATGTGAAAAGTCTTGTTTGGTAACCATCTATGCTTAAATACAAAA
  >chr4:93023010-93023198 - E=3e-101
ATTAAATCTAATTATTTTGAAATTTCTTTGCTTCCCCAAATATCAAAAACACAAGATTTAAGCAAGCTCCAAAATTGCTCAATAAATTT

|GCAGCCTACAGCAAGTGCTCAGCCA|GCTC|AATAAATTTTTTCTGTGCCAATTCTTATTGCATAAGCTGCTTTCCTCTCTGGCTACT
  >chr4:93022356-93022385 - E=1e-05 >chr4:93022833-93023023 - E=2e-102
AACCCTACACCTTAATCACATAAACTCTGAAACTAAGACTCATGTCTTAAGAAGTATTTAAACAACACTAATTTCTAAAATAGGTGATG

TAATGCAGTTATTCAGAAGGAAAAATATCTCAAAGACAT|T|AACACATGGCAGCCTACAGCAAGTGCTCAGCCAGCTCCCTGCCAGAG
                                        >chr4:93022095-93022394 - E=6e-168
AAACATGCCTTATCCACAGGTTATAGAACAAACACAGATGCAGCAAAATGAGTCACCAGAGGAAAACAAAAGCCTAAATTGAAGGTAAA

TGAAGCTTTGTGCACATGCATGACAAGAAATCCATTGAGCAAAACTAATTTTATATCTGTGCCAGAATGGCTGTCTTTTATGTTGGGAA

TAGGAAACAGGAGGGAAAGGAAAGTCACATAATAGTAGACCTTGAAGTGTACTAAGATAGAGAATACCTTCTG
```
